# Cowden Syndrome: A Rare Cause of Intestinal Polyposis

**DOI:** 10.7759/cureus.64838

**Published:** 2024-07-18

**Authors:** Hasan M Isa, Zahra S Mohamed, Zahra H Isa, Maryam Y Busehail, Zahra A Alaradi

**Affiliations:** 1 Department of Pediatrics, Arabian Gulf University, Manama, BHR; 2 Department of Pediatrics, Salmaniya Medical Complex, Manama, BHR

**Keywords:** bahrain, cancer predisposition, multiple hamartoma syndrome, gastrointestinal polyposis, macrocephaly, pten gene mutation, cowden syndrome

## Abstract

Cowden syndrome (CS) is a rare autosomal dominant genodermatosis disorder. This disease is characterized by the development of several hamartomata lesions in a variety of tissues from all three embryonic layers. The most well-known hamartomata are those of the gastrointestinal system, which represent one of the major criteria for the diagnosis of CS. Yet, the most frequent initial presenting symptom of the disease is thought to be mucocutaneous symptoms such as trichilemmomas, acral keratosis, and oral papilloma. Early diagnosis and management are essential to improving the quality of life for patients with CS as this disorder predisposes them to cancers such as thyroid, breast, gastrointestinal, and endometrial cancers. This report presents a rare case of CS in a Bahraini child who presented with macrocephaly and had numerous intestinal polyposis. Genetic testing using whole exome sequencing confirmed the diagnosis, identifying a pathogenic de novo phosphatase and tensin homolog gene (*PTEN*) variant (Chr10 NM_000314.8: c.17_18del p.(Lys6Argfs*4)) in a heterozygous state. This variant has been confirmed by Sanger sequencing.

## Introduction

Cowden syndrome (CS), also known as multiple hamartoma syndrome, is a rare genodermatosis of autosomal dominant inheritance originally identified in 1963 [1,2]. It is caused by a germline mutation of the phosphatase and tensin (*PTEN*) homolog gene detected on chromosome 10 [1,2]. Mutations in the *PTEN* gene are responsible for CS and other *PTEN* hamartomatous tumor syndromes (PHTS) [2]. *PTEN* is a tumor suppressor gene that manifests with hamartomatous growths involving all three embryonic origins [3]. CS is primarily a disease of young adults (second and third decades) but can occur at any age between four and 75 years [4].

Although the expression of the disease is variable, mucocutaneous lesions are pathognomonic [5]. These lesions usually represent the first sign to be identified in patients with CS [5]. Moreover, the most commonly identifiable feature is the extensive proliferation of multiple hamartomas all over the body especially in the gastrointestinal (GI) tract [6]. Since CS is a well-known condition that predisposes to several types of malignancy, it is predicted that 39.1% of affected persons have been diagnosed with at least one malignant tumor [6]. As an exception, the risk of GI cancers is not well characterized, as GI polyposis is not known to increase the cancer risk [7,8].

Here, we are presenting a Bahraini male child who had macrocephaly along with numerous intestinal polyposis and the diagnosis of CS was confirmed by genetic testing.

## Case presentation

**Figure 1:**
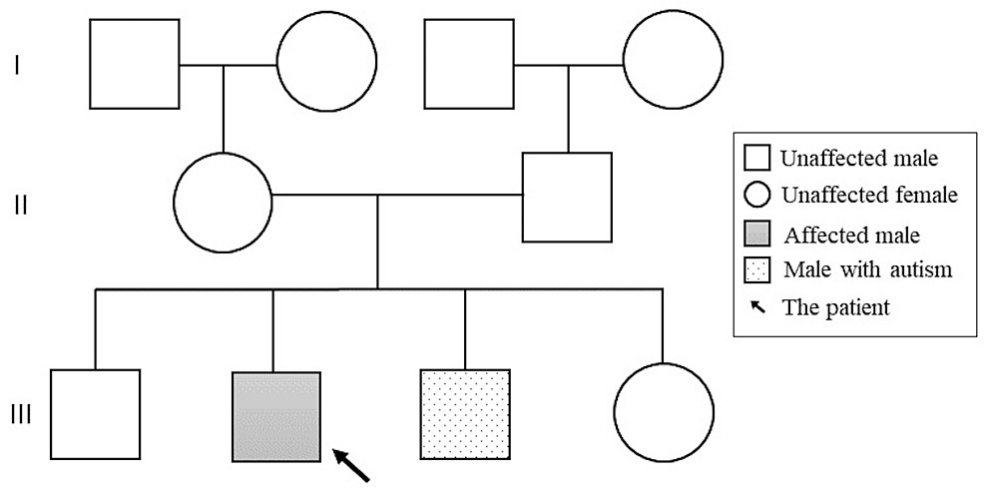
Family pedigree of a child with Cowden syndrome. Image credits: Hasan M. Isa, Zahraa S. Mohamed, Zahra H. Isa, Maryam Y. Busehail, Zahraa A. Alaradi.

This patient is a Bahraini male child born at term via normal vaginal delivery, with a birth weight of 3.4 kilograms, to non-consanguineous parents. He had three siblings, two brothers and one sister. His youngest brother was diagnosed with autism spectrum disorder (ASD) (Figure 1).

Prenatal ultrasounds revealed the patient had a large-sized head across three different routine antenatal visits. During the postnatal examination, his head circumference was 45 cm (>99th percentile) indicating macrocephaly. Despite this finding, the patient was stable. He was discharged and given a follow-up appointment at the pediatric outpatient clinic.

**Figure 2:**
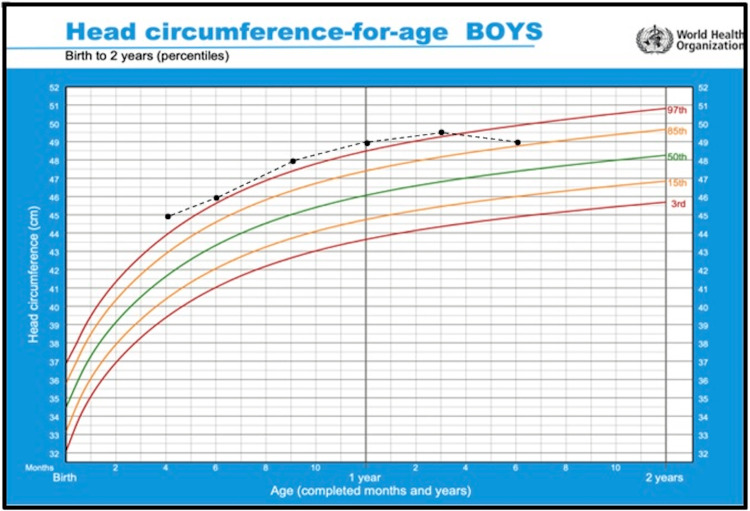
Head circumference chart showing macrocephaly in a child with Cowden syndrome.

At the age of four months, the patient's head circumference was stable at 45 cm (>99th percentile), and he was showing normal other growth and developmental milestones. At six months of age, his head circumference was 46 cm (99th percentile) and his weight was 6.6 kilograms (5th percentile) (Figure 2).

**Figure 3:**
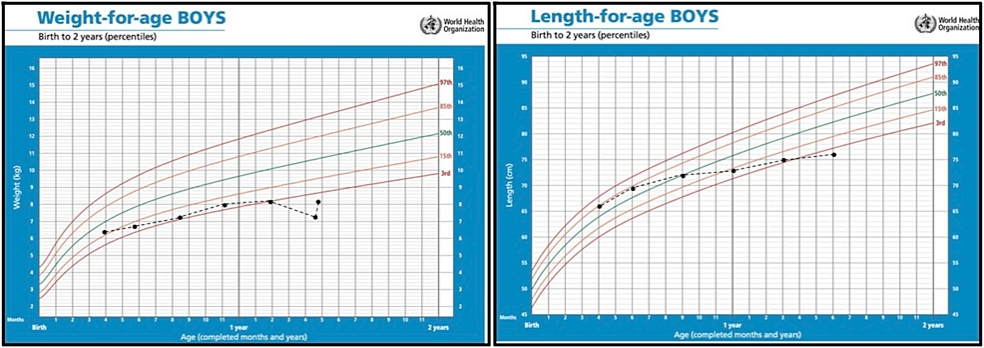
Growth chart showing the weight and height growth of a child with Cowden syndrome.

At the age of nine months, his head circumference was 48 cm (>99th percentile) and he started to develop low muscle tone for which he was referred for physiotherapy. Later, his hypotonia started to affect his ability to climb the stairs. The patient's weight and height growth are shown in Figure 3.

**Figure 4:**
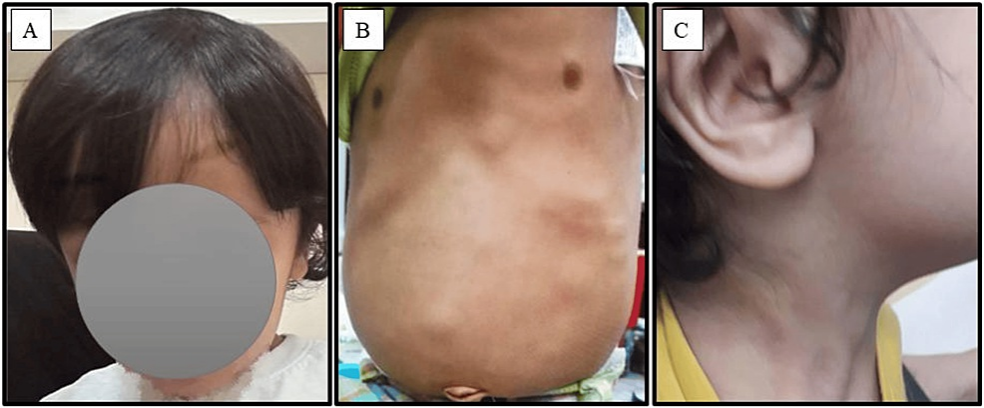
A child with Cowden syndrome. The child's pictures show macrocephaly (A), multiple abdominal nodules (B), and hyperpigmentation mainly affecting the neck (C).

At the age of two years, his head circumference was 52 cm (>99th percentile) (Figure 4A).

**Figure 5:**
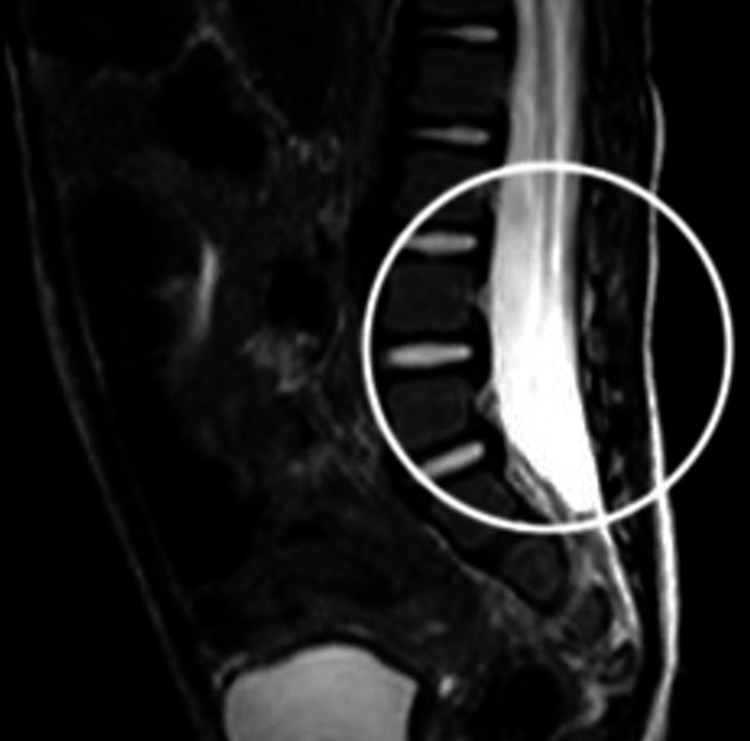
T2 weighted sagittal view of magnetic resonance imaging of the lower spine showing incidental finding of dural ectasia (white circle) in a child with Cowden syndrome.

In addition, abnormal speech development was detected. Accordingly, a computerized tomography (CT) brain scan was performed at a private hospital which revealed an empty sella, prompting the patient to be referred to a pediatric neurologist, endocrinologist, and metabolic experts. During the initial laboratory investigations, complete blood count, iron profile, renal function tests, liver function tests, celiac screen, thyroid function tests, cortisol level, vitamin D, and sweat chloride test were performed. Except for iron deficiency anemia, they were all unremarkable. Tests for tandem mass spectrometry, very long chain fatty acids, and urine organic acids were performed to exclude inborn metabolic abnormalities, and all were negative. Moreover, pituitary gland functions were evaluated and found to be normal. Magnetic resonance imaging (MRI) brain and spine was normal apart from an incidental finding of a widening of the dural sac affecting the whole lumbar area, indicating dural ectasia, with no injury to the posterior vertebral bodies (Figure 5).

At three years of age, the patient remained hypotonic but had completely normal deep tendon reflexes, and his musculoskeletal examination revealed no abnormalities. Abdominal distention was noted but with no hepatosplenomegaly. Due to the weight faltering, the patient was assessed by a dietician and a gastroenterologist. At the age of three years and four months, as part of the investigations ordered by the gastroenterologist, abdominal ultrasonography was done which showed two superficial cutaneous hamartomas.

This finding with the macrocephaly and intellectual disabilities of the patient necessitated genetic testing. Whole exome sequencing revealed a heterozygous pathogenic variant which was identified in the *PTEN* gene, Chr10 NM_000314.8 (GRCh37): g.89624243_89624244del, c.17_18del p.(Lys6Argfs*4) that shifted the reading frame starting at codon 6. The new reading frame ends in a stop codon 3 positions downstream. This variant was confirmed by Sanger sequencing. In the parents' genetic testing, the mutation was negative (not detected), thereby it was a de novo mutation. Additionally, his younger brother was investigated, and the variant was not identified. Once the diagnosis of CS was confirmed, the patient was given follow-up appointments with the gastroenterologist and dermatologist for regular monitoring of his condition. 

**Figure 6:**
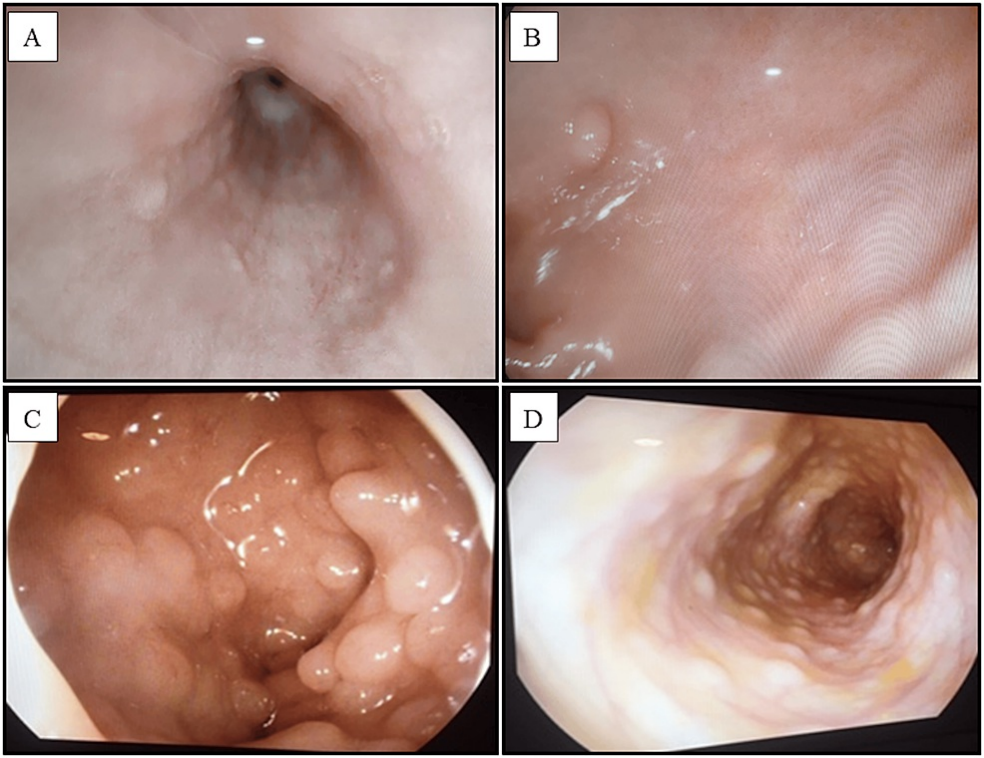
Upper gastrointestinal endoscopy and colonoscopy of a child with Cowden syndrome. Upper gastrointestinal endoscopy showing small-sized esophageal polyps (A), a gastric polyp (B), and multiple duodenal polyps (C); and colonoscopy showing scattered colonic polyps (D).

At the age of three years and six months, the patient underwent a barium swallow study, an upper GI endoscopy, and a colonoscopy. Barium swallow showed minimal gastroesophageal reflux (GER). The GI endoscopy showed a few small-sized raised mucosal polyps in the middle esophagus, two small-sized polyps in the stomach, multiple duodenal polyps, and scattered colonic polyps (Figure 6).

Biopsies were taken from the polyps for histopathological examination and the patient was referred to an oncologist to assess the risk of developing malignancies. The initial assessment was unremarkable as all hamartomas were found to be benign overgrowths. Moreover, abdominal ultrasonography was repeated and showed no malignant growth in the abdomen. The risk of developing malignancy and the need for further assessment was explained to the parents. 

At the age of three years and 10 months, the patient’s examination revealed multiple abdominal nodules (Figure 4B), as well as a suspicion of retractile testicles, which required referral to a pediatric surgeon. A surgical examination showed a left retractile testis that comes back easily and stays in the scrotum along with the normal right testicle. Repeated abdominal wall ultrasonography showed multiple well-defined subcutaneous fat echogenic lesions in the anterior and left lateral abdominal wall, indicating lipomas. The patient was also reviewed again by the dermatologist as a part of follow-up for the manifestations of CS and noted no new skin changes. However, at the age of four years and seven months, he started to manifest hyperpigmented patches on the back and lichenification on the dorsum of the hands and fingers. Dexapanthenol cream was prescribed to decrease the pigmentations with Vaseline ointment for lichenification. Further follow-up visits showed small areas of hyperpigmentation mostly affecting the face and neck (Figure 4C).

At seven years of age, the GI endoscopy was repeated, which showed multiple GI polyposis, but no signs of malignancy were detected on histology. The patient's language development was delayed as he wasn’t talking, and he was using his mother as a tool to express himself. Speech assessment showed that the patient was only able to say his first name and a few words. Furthermore, he showed autistic-like behaviors, such as trouble maintaining eye contact and a lack of interest in peer play. This necessitated further investigations and follow-up with a psychiatrist until a definitive diagnosis of ASD was made in the same year. The patient continues to follow up in multiple subspecialty clinics including pediatric gastroenterology, oncology, and dermatology. 

## Discussion

**Figure 7:**
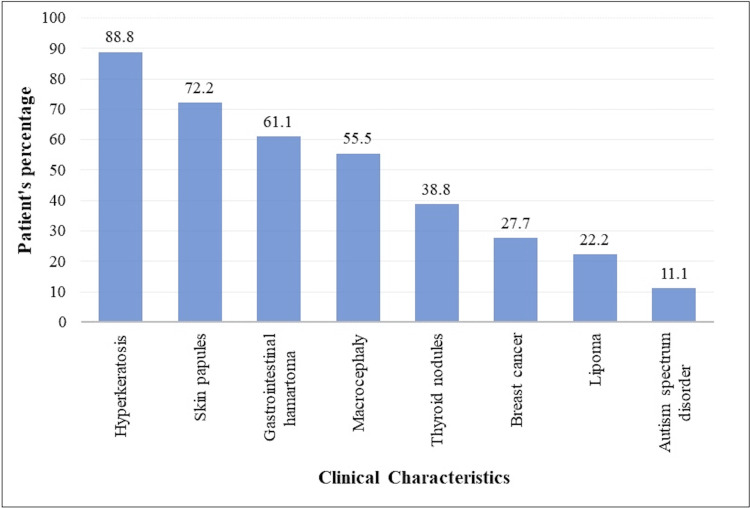
Clinical features of patients with Cowden syndrome. Summary of 15 previously reported case reports including the current report [1,3-16]. Image credits: Hasan M. Isa, Zahraa S. Mohamed, Zahra H. Isa, Maryam Y. Busehail, Zahraa A. Alaradi.

This is the first identified case of CS in Bahrain. This condition affects one in every 200,000 people [9]. Globally, there are less than 500 reported cases with the same syndrome, with more than 200 cases diagnosed only in Japan [10,11]. The disease's wide phenotypic diversity might complicate the diagnosis [1]. Clinical presentations of CS are summarized in Figure 7.

CS is primarily an autosomal dominant disease caused by a mutation in the *PTEN* suppressor gene, with incomplete penetrance and variable expressivity [4,8,12]. De novo mutations account for 45% of cases while a much smaller number is reported to be due to* PTEN* mosaicism [2].

For the diagnosis of CS, the clinical criteria of the international Cowden consortium are chiefly used [8,11]. Our patient was diagnosed with three major criteria which are macrocephaly, GI hamartomas, and acral keratosis fulfilling the criteria for the diagnosis. Moreover, the presence of two minor criteria (lipoma and ASD) supports the diagnosis. Despite the fact that a diagnosis based on physical findings can be used in some cases, it has been reported to be extremely difficult, especially in early life [11]. 

Although *PTEN* mutations are not included in the CS diagnostic criteria, they are found in 80% of patients with CS, indicating their importance in CS surveillance [11]. *PTEN* is a tumor suppression gene that is commonly deleted at chromosome 10q23 [5,12]. It is known that mutations in exons 5, 7, and 8 can truncate the protein or change the phosphatase activity [12]. In our patient, whole exome sequencing revealed a heterozygous pathogenic variant, which was identified in the *PTEN* gene, Chr10 NM_000314.8 (GRCh37): g.89624243_89624244del, c.17_18del p.(Lys6Argfs*4). According to the Human Gene Mutation Database (HGMD) Professional 2018.2, this variant was previously described as a disease-causing CS [17]. ClinVar lists this variant as pathogenic (clinical testing, variation ID: 231649) [18].

Genetic testing was also reported by eight out of the 15 reviewed studies, with only six of them providing the whole genome sequence. Porto et al. showed a *PTEN* mutation in exon 8 as a heterozygous deletion of nucleotide 968 [5]. Patini et al. showed a *PTEN* germline mutation [c. 697 C<T (R233X)] [6]. Ha et al. revealed a nonsense *PTEN* mutation (NM_000314.4 c.633C > A) in exon 6 as a heterozygous transition of C to A at nucleotide 633, making a stop codon (p. Cys211*) [8]. Umemura et al. showed a *PTEN* point mutation in exon 8, as it was of C to T at codon 1003 [11]. Son et al. showed a *PTEN* mutation (c.388C>T (p. Arg130*)) in exon 5 in a heterozygous state [12]. During their investigation of a child with ASD, Gruhl et al. unintentionally discovered that the father possessed a *PTEN* mutation at base 176, c.176C>G in exon 3 with C to G alteration converting amino acid number 59 from a serine to a stop codon, p.S59X [9]. Five of the studies relied solely on clinical criteria for diagnosis [3,4,7,14,15]. Moreover, one case was confirmed using both clinical and genetic criteria, although no sequence was provided [10]. 

Macrocephaly is one of the major criteria for diagnosis of CS and has been reported in 80% of patients [5,10]. Differential diagnosis between CS and other conditions such as basal cell nevus syndrome and neurofibromatosis type 1 is valuable in these cases as macrocephaly is progressive in the first years of life [1]. Some CS cases have reported an association between CS and Lhermitte-Duclos disease, with marked improvement in the prognosis if associated with early MRI imaging [13]. 

Mucocutaneous manifestations are reported to be present in 90-100% of CS cases, which represent a prominent feature for diagnosis and is known to be the disease’s earliest symptom [12,14]. The manifestations include trichilemmomas, acral keratosis, and oral papillomas, which mostly give a “cobblestone appearance” [1,2]. In the present case, skin affection was in the form of hyperpigmentation and acral keratosis which were noted in the third and seventh years of age, respectively. However, most of the reported dermatological symptoms are predominantly present in the second and third decades [2,12]. Further dermatological signs included macular pigmentations of the glans penis, which is a major prerequisite for CS diagnosis, present in 46-54% of male patients [1,2]. Lipomas and testicular lipomatosis are minor dermatological criteria that account for 40% of CS cases [2].

CS is a classic hamartomatous polyposis condition characterized by different polyp forms that develop across the entire GI tract, with an incidence reaching up to 71% [1,2,7,14]. The pathological spectrum of GI polyps includes hamartomata, lipomatous, juvenile, lymphomatous, hyperplastic, inflammatory, and only occasionally adenomatous [7,12]. As in our case, the diagnosis was confirmed with upper GI endoscopy and colonoscopy, which are essential for the diagnosis [12]. Esophageal polyposis was reported in 85.7% of cases, in which histopathology is mostly suggestive of glycogenic acanthosis [11]. Colonic polyposis may be the first sign of CS with a 16% probability of developing colorectal cancer [1,2,14]. 

Cognitive affection has been reported in individuals with CS, ranging from undetectable impairment to the development of ASD or mental retardation [2]. Studies showed that only 20% of CS cases had an association with mental retardation, and only a handful reports with ASD association [2,9]. The association between ASD and macrocephaly is present in our case, in which MRI studies describe the association as a result of overgrowth of the white matter [9]. 

Thyroid abnormalities are a major criterion for diagnosing CS, accounting for 67% of cases [10,12]. Cases are presented mainly with benign findings such as multinodular goiter and multiple thyroid adenomas, with a 12% possibility of developing cancer exclusively follicular or papillary adenocarcinoma [2,5,9,10]. Thyroid involvement was not detected in our patient. This can be attributed to the patient's young age, and he may experience additional symptoms in the future. Moreover, we were unable to detect any malignant disease in our patient up till now. However, early diagnosis of complications related to CS is required as malignant tumors have been reported in at least 30-40% of CS patients [1,11]. The risk of cancer is primarily associated with the breast, endometrium, thyroid, brain, and colon, with a small minority of cases having a risk of melanoma (less than 6%) [1-3]. The risk of GI cancers in these patients is not well characterized as GI polyposis is not known to increase the cancer risk [3,7,19]. These findings highlight the importance of routine screening and follow-up to enable the early identification of any malignant alteration [8,16]. 

## Conclusions

CS is a rare genetic disease characterized mainly by the development of hamartomas. These growths are commonly found in the skin and mucous membranes, but they can also occur in the intestine and other parts of the body. Macrocephaly, which can be detected antenatally, might be the first clue of CS. Patients with CS should be diagnosed early by genetic testing, which should be followed by screening for cancer growth in order to aid patients and their families in seeking earlier medical guidance that can extend their life expectancy. Further studies focusing on the phenotype-genotype correlation of this disease are needed.
